# Melioidosis with Mediastinal and Cardiac Invasion

**DOI:** 10.4269/ajtmh.26-0511

**Published:** 2026-11-03

**Authors:** Deng-Wei Chou, Fu-Chi Fang, Shu-Ling Wu

**Affiliations:** ^1^ Department of Critical Care Medicine, Tainan Municipal Hospital (Managed by Show Chwan Medical Care Corporation), Tainan City, Taiwan;; ^2^ Department of Nursing, Chung Hwa University of Medical Technology, Tainan City, Taiwan;; ^3^ Department of Cardiovascular Surgery, Tainan Municipal Hospital (Managed by Show Chwan Medical Care Corporation), Tainan City, Taiwan;; ^4^ Department of Long-Term Care and Management, Chung Hwa University of Medical Technology, Tainan City, Taiwan

A 63-year-old man with a history of type 2 diabetes mellitus presented to our emergency department with intermittent fever that had lasted 3 weeks. He is Taiwanese, lives in southern Taiwan, and had no overseas travel history. Physical examination revealed a body temperature of 38.3°C, a heart rate of 98 beats/minute, a respiratory rate of 24 breaths/minute, and blood pressure of 90/52 mm of Hg. Distant and muffled heart sounds and distended neck veins were identified. Laboratory examinations yielded a white blood cell count of 15,700 cells/mm^3^ with 86% neutrophils, and a C-reactive protein level of 27 mg/dL (reference: <0.3 mg/dL). Chest radiography (Figure 1A) revealed a bulging mass in the right hilar area along with cardiomegaly. Chest computed tomography (CT) (Figures 1B and 2) revealed a mediastinal mass measuring approximately 4.6 cm, multiple enlarged lymph nodes, and massive pericardial effusion. There were no other multiorgan abscesses, septic emboli, or pulmonary embolism from CT. Echocardiography indicated considerable pericardiac effusion with early diastolic collapse of the right ventricle, suggestive of cardiac tamponade. An emergent pericardial window was performed for the patient’s cardiac tamponade. A total of 300 mL of noncoagulable and reddish fluid was drained. To establish a diagnosis, surgery was performed to remove the mediastinal mass and enlarged mediastinal lymph nodes. Pathological examination (Figure 3) of resected mediastinal mass and pericardium showed necrotizing inflammation. Pathological examination of resected lymph nodes revealed acute lymphadenitis. There were no malignant cells or acid-fast bacilli in the pericardium, mediastinal mass, or enlarged lymph nodes. Later, blood, pericardial fluid, mediastinal mass, and lymph nodes cultures were all positive for *Burkholderia pseudomallei*, with identical antimicrobial susceptibility. Antimicrobial susceptibility testing using minimum inhibitory concentration assay showed the pathogen was susceptible to imipenem, ceftazidime, trimethoprim/sulfamethoxazole (TMP-SMX), amoxicillin-clavulanate, and doxycycline. Once a diagnosis of melioidosis was established, the patient was successfully treated with intravenous ceftazidime for 2 weeks (2,000 mg every 8 hours) followed by oral TMP-SMX therapy for 3 months (240/1,200 mg every 12 hours). No evidence of recurrent melioidosis was detected at the 1-year follow-up.

**Figure 1.**
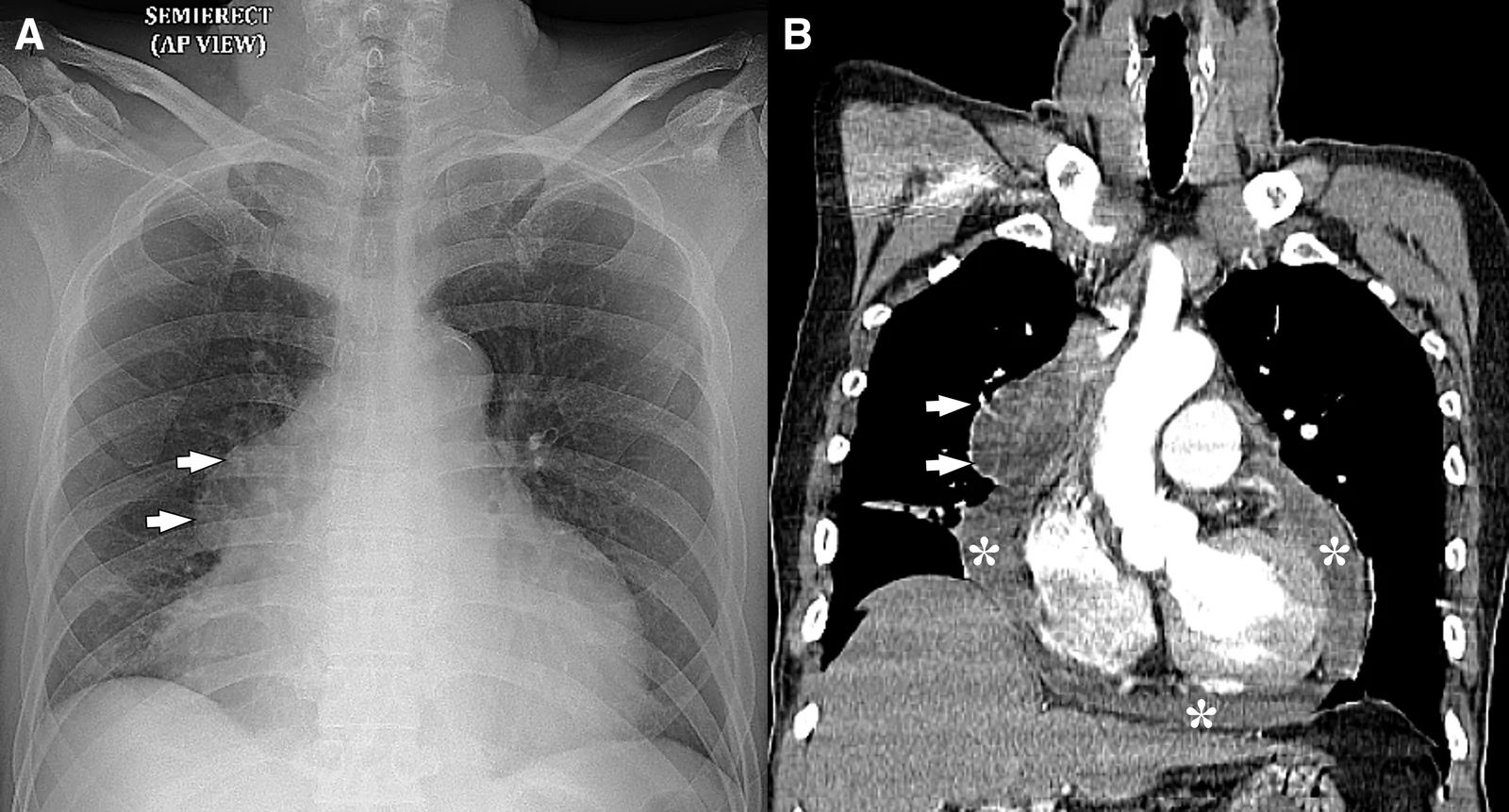
(**A**) Chest radiography (AP view) revealing a bulging mass in the right hilar area (white arrows) along with cardiomegaly. (**B**) Contrast-enhanced computed tomography image of the chest (sagittal view) revealing a mediastinal mass measuring approximately 4.6 cm (white arrows) and massive pericardial effusion (asterisks).

**Figure 2.**
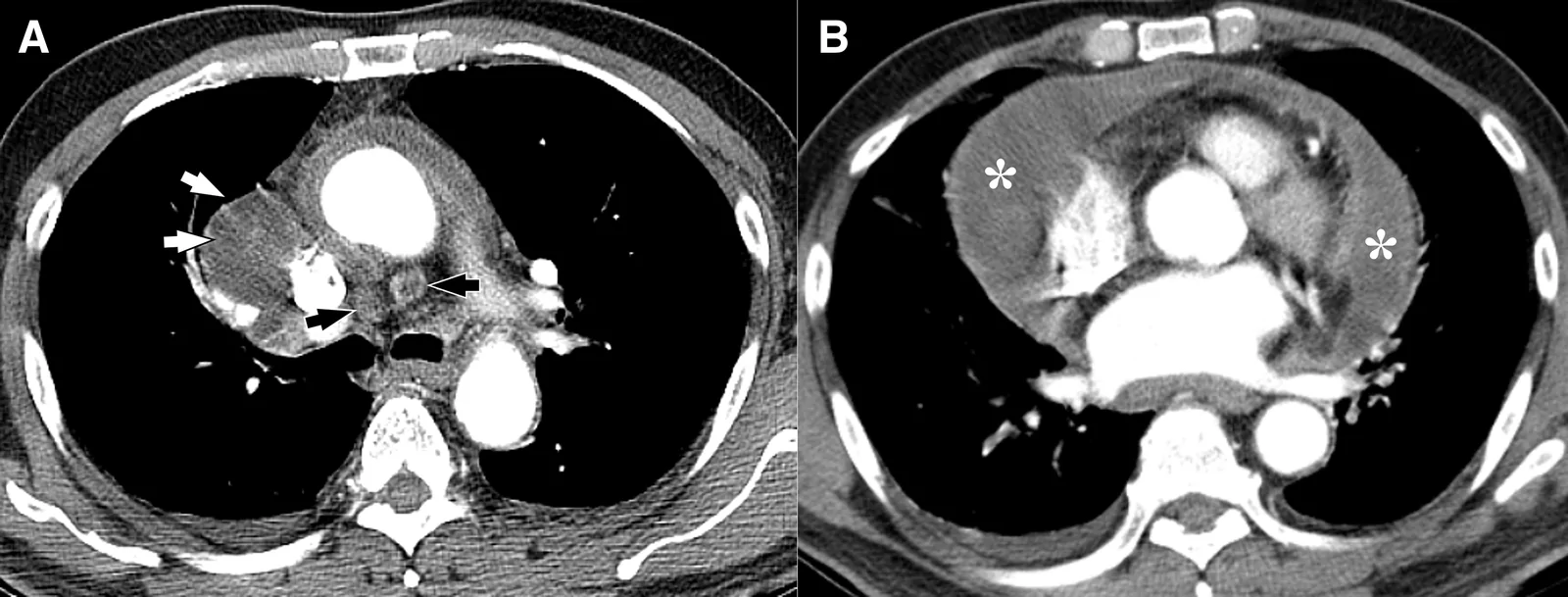
(**A**) Cross-sectional view showing a mediastinal mass (white arrows) accompanied by multiple enlarged lymph nodes with central low attenuation and peripheral rim enhancement (black arrows). (**B**) Cross-sectional view revealing massive pericardial effusion (asterisks).

**Figure 3.**
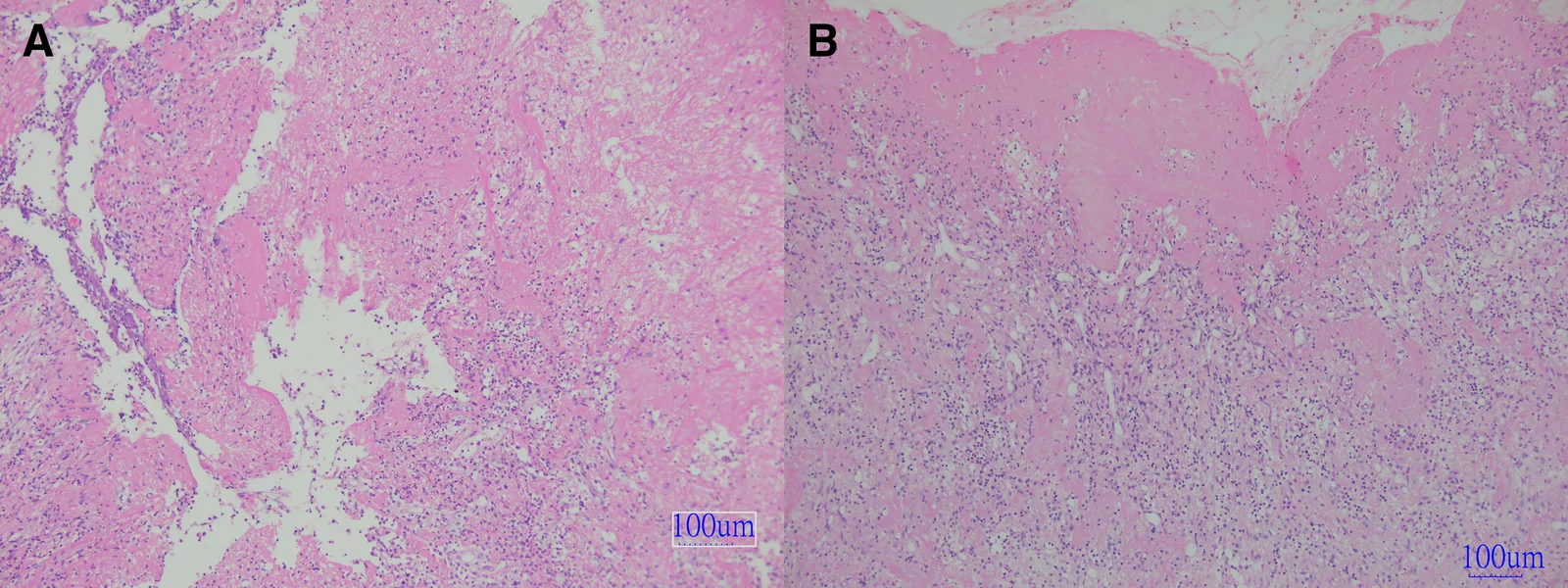
Pathologic findings of (**A**) mediastinum mass showing necrotic fibrinous inflammation with mixed inflammatory cells infiltration. (100×) (**B**) pericardium showing necrotizing inflammation of pericardium with fibrinous exudate and mixed inflammatory cells infiltration. (100×)

Melioidosis is a disease caused by the environmental pathogen and Gram-negative bacillus *B. pseudomallei*. Melioidosis is becoming endemic to tropical regions worldwide and is spreading to nonendemic areas.^1^ Melioidosis primarily affects the lungs, genitourinary tract, and musculoskeletal system, but it rarely involves the mediastinum and heart.^2^ Melioidosis is a cogent mimicker. Apart from tuberculosis and malignancy, melioidosis should always be considered as one of the differential diagnoses of cardiac tamponade, especially in the areas where tuberculosis and melioidosis are endemic.^3^^,^^4^ Establishing a definitive diagnosis requires focusing on pericardial pathology. In a systemic review of *B. pseudomallei* with cardiac involvement, antimicrobial therapy included an initial induction therapy with ceftazidime or a carbapenem ± TMP-SMX, followed by eradication therapy with TMP–SMX in most patients.^5^

Melioidosis has become endemic in southern Taiwan.^2^ We present a case of domestic melioidosis occurring simultaneously with a mediastinal mass, mediastinal lymphadenopathy, pericarditis, and cardiac tamponade. Currently, limited information is available regarding this rare and complicated disease. This report aims to raise awareness among clinicians regarding the complex symptoms of melioidosis, with the ultimate goal of improving patient outcomes.

## References

[1] Raja NS Ahmed MZ Singh NN, 2005. Melioidosis: An emerging infectious disease. J Postgrad Med 51(2): 140–145. 16006713

[2] Chou DW Chung KM Chen CH Cheung BM, 2007. Bacteremic melioidosis in southern Taiwan: Clinical characteristics and outcome. J Formos Med Assoc 106(12): 1013–1022. 10.1016/S0929-6646(08)60077-7 18194907

[3] Chetchotisakd P Anunnatsiri S Kiatchoosakun S Kularbkaew C, 2010. Melioidosis pericarditis mimicking tuberculous pericarditis. Clin Infect Dis 51(5): e46–e49. 10.1086/655699 20645861

[4] Chung HC Lee CT Lai CH Huang CK Lin JN Liang SH Lin HH, 2008. Non-septicemic melioidosis presenting as cardiac tamponade. Am J Trop Med Hyg 79(3): 455–457. 18784243

[5] Velusamy R Muhi S, 2020. Melioidosis and the heart: A systematic review. Trop Med Infect Dis 5(3): 121. 10.3390/tropicalmed5030121 PMC7558958 32717859

